# The Value Proposition of Coordinated Population Cohorts Across Africa

**DOI:** 10.1146/annurev-biodatasci-020722-015026

**Published:** 2024-08-01

**Authors:** Michèle Ramsay, Amelia C. Crampin, Ayaga A. Bawah, Evelyn Gitau, Kobus Herbst

**Affiliations:** 1 Sydney Brenner Institute for Molecular Bioscience, Faculty of Health Sciences, University of the Witwatersrand, Johannesburg, South Africa; 2 Malawi Epidemiology and Intervention Research Unit, Lilongwe, Malawi; 3 Regional Institute for Population Studies, University of Ghana, Accra, Ghana; 4 African Population and Health Research Center, Nairobi, Kenya; 5 Africa Health Research Institute, Durban, South Africa; 6 South African Population Research Infrastructure Network, Department of Science and Innovation and South African Medical Research Council, Durban, South Africa

**Keywords:** population cohorts, research, health, Africa, value proposition

## Abstract

Building longitudinal population cohorts in Africa for coordinated research and surveillance can influence the setting of national health priorities, lead to the introduction of appropriate interventions, and provide evidence for targeted treatment, leading to better health across the continent. However, compared to cohorts from the global north, longitudinal continental African population cohorts remain scarce, are relatively small in size, and lack data complexity. As infections and noncommunicable diseases disproportionately affect Africa’s approximately 1.4 billion inhabitants, African cohorts present a unique opportunity for research and surveillance. High genetic diversity in African populations and multiomic research studies, together with detailed phenotyping and clinical profiling, will be a treasure trove for discovery. The outcomes, including novel drug targets, biological pathways for disease, and gene-environment interactions, will boost precision medicine approaches, not only in Africa but across the globe.

## 1 Burden Of Disease In The Context Of African Populations And The Need For Research Cohorts

Developing research and surveillance cohorts across Africa to reflect the changing demographic, health, and socioeconomic statuses of populations is complex. Africa is one of the most diverse continents worldwide, not only in terms of ethnic composition, its general demography, culture, and economy, but also in terms of its political and social organizational systems (1) and levels of inequality (2). With more than 3,000 ethnic groups and over 2,000 languages spoken, Africa’s population is estimated to be around 1.4 billion, with approximately 1.2 billion residing in sub-Saharan Africa (SSA) (3). Africa is the continent with the fastest-growing population, with the population of SSA expected to almost double, to more than 2 billion, by 2050 (3). Although it has a young population demographic, Africa is also experiencing population aging from a current 5.5% of the population aged 60+ years old rising to a projected 8.7% by 2050 (4).

Africa’s political history has been checkered. Starting in the 1960s, many countries on the continent had gained political independence and ushered in constitutional rule. However, shortly after, several countries entered a period of turmoil. In addition to military takeovers, particularly in West and Central Africa, civil and armed conflicts and wars; religious and ethnic conflicts; and, more recently, terrorist activities, especially in the Sahel region, became a common feature of the continent (5). This has led to loss of lives and property, destruction of infrastructure, disruption in economic growth, and displacement of large sections of populations, resulting in large groups of refugees in many parts of the continent, stifling economic development (6) and impacting health and the attainment of the Sustainable Development Goals (SDGs) (7).

Meeting the global SDGs in Africa, especially those addressing poverty alleviation, is critical if the health-related goals are to come within reach of individual countries. This requires a deep understanding of the complexity of exposures, health trajectories, and outcomes, frequently captured in high-income settings through linkage between routine health data and denominator data. The lack of electronic health records and reliable vital event registration across most of Africa, the inadequate skills base, the low ratio of health-care professionals to persons in the populations they serve, low universal health coverage (UHC), and low health system functionality (8, 9) will continue to plague advances toward global goals for improved health in African settings.

Africa is witness to the collision of infectious disease pandemics with the rising prevalence of noncommunicable diseases, leading to frequent multimorbidity across the spectrum from urban to rural communities. Patterns of multimorbidity, conditions included, diagnostic definitions, and study methods differ across studies, preventing comparisons but also obscuring the full health burden. A recent literature review on multimorbidity in African-ancestry populations contrasted findings from studies in Africa to those from the diaspora, demonstrating that although cardiometabolic diseases (CMDs) dominate in both settings, infectious diseases are more commonly included in studies performed on the continent (10). The inclusion of infectious diseases is more prominent in different lower- and middle-income country (LMIC) settings, including those in Asia (10, 11). A common theme across three reviews on multimorbidity (10–12) is the call for robust methods and approaches for population-level data collection for a wide range of chronic conditions and population representations. Such an approach would be supported by population cohorts across Africa.

The global rise in genomic studies to inform precision medicine, leading to more effective diagnoses, interventions, and health outcomes, is not reflected in Africa. African populations have the greatest genetic diversity (13), with only a subset of variants leaving the continent in major waves of out-migration between 70,000 and 35,000 years ago (14–16), leaving vast unexplored variation in African populations. The poor representation of African populations in large genomic studies has been lamented by many over several decades, yet the divide is widening as Eurocentric studies with large cohorts continue on an exponential rise (14, 17–19), becoming the biggest influencer of discovery research in human health.

**Figure 1:**
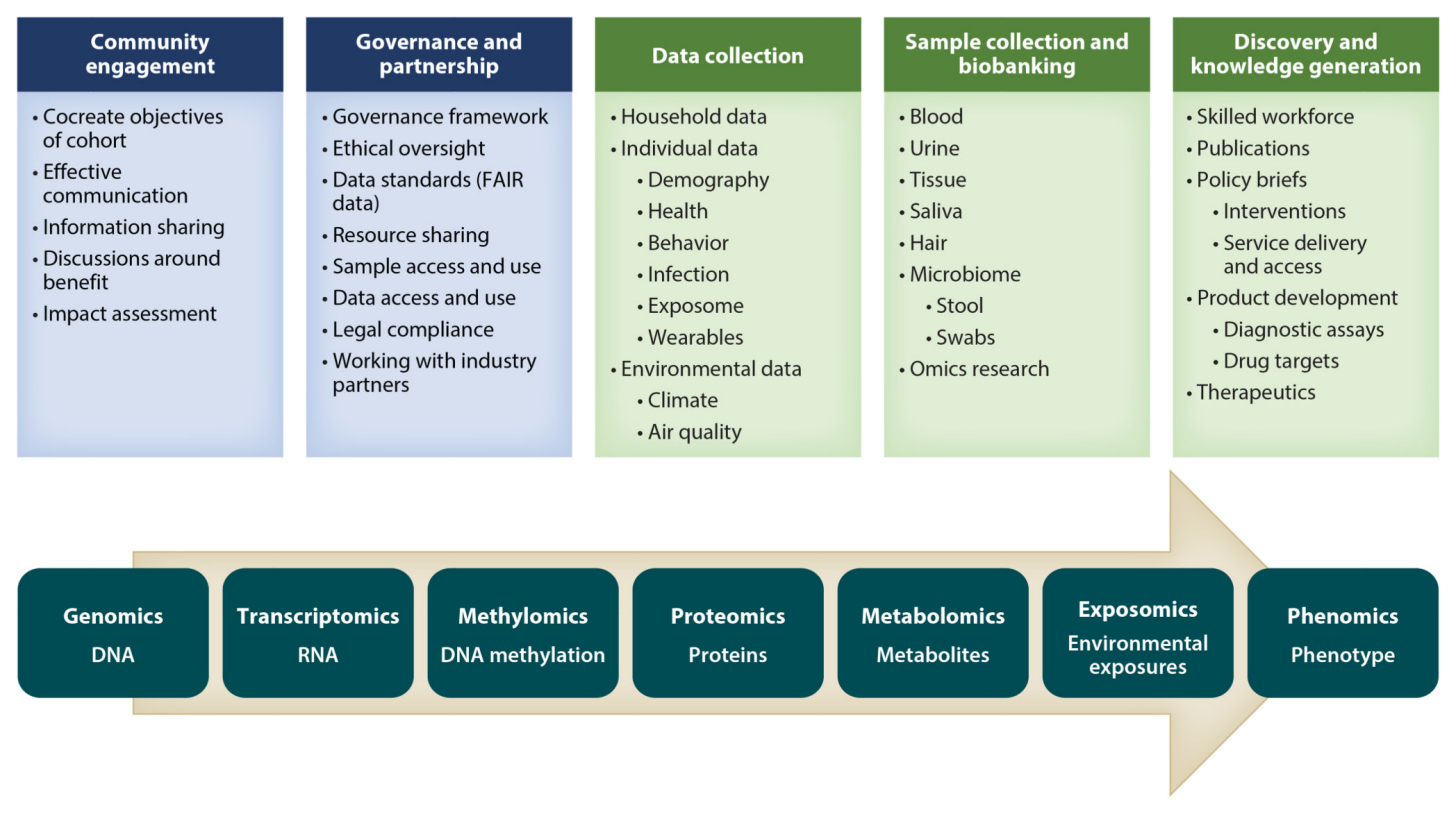
Building cohorts and preparing for omics research to enhance scientific discovery: points to consider. Abbreviation: FAIR, findable, accessible, interoperable, and reusable.

Building sustainable research and surveillance cohorts, linked to rich biorepositories in countries across Africa, is an imperative. Such cohorts will need to be built on the pillars of effective and extensive community engagement and good governance frameworks, with informed consent models that are compatible with data and sample sharing for future research, including omics research (Figure 1). The phenotypic and omic data will inform and promote discovery and evidence-based policies and interventions to improve the future health of Africa’s diverse populations and will impact other global populations. This review sketches the landscape across the continent and identifies key barriers and obstacles while emphasizing the power of data from large longitudinal population cohorts.

## 2 Cohort Models

Epidemiologists define a cohort (20, 21) as a group of individuals, identified and described in a specific manner, who have been followed for more than one wave of data collection. They could be population cross-sectional cohorts or cohorts of individuals selected on the basis of having an identifiable disease or condition. In this review, we focus on population cohorts rather than disease-specific or hospital cohorts. Population cohorts are agnostic to disease and by definition would include individuals with common diseases (and a smaller number with rare diseases), reflecting the prevalence of these diseases in the study population.

The nature and application of a cohort is defined by several factors, including selection criteria (e.g., a random cross-section of the defined population, a specific age stratum, including men and women, births occurring in a specific time period and setting), cohort size, the amount and complexity of data collected (including demographic, social, behavioral, physiological, health, and genetic), the length of time the cohort has been followed, and whether biological samples have been collected for future use. Table 1 highlights the strengths and limitations of different types of cohorts in an African setting.

Africa has dozens of cohorts, most of which are located within a single country. However, there are several multicountry cohorts connected through networks such as the Prospective Urban and Rural Epidemiology study (22), the Analysing Longitudinal Population-Based HIV/AIDS Data on Africa Network (23), the International Network for the Demographic Evaluation of Populations and Their Health in Low- and Middle-Income Countries (INDEPTH) Health and Demographic Surveillance System (HDSS) Centres (24), and the Human Heredity and Health in Africa (H3Africa) Consortium (25), each with different models in operation. The purpose and design of cohorts and networks differ, and the depth and breadth of data collection differs, with limited data interoperability across cohorts and sometimes even within networks.

Long-term cohorts are important for understanding the burden and distribution of important health conditions and relevant exposures, including behavioral, social, genetic, and environmental data, some of which may be specific to the setting. They should be able to inform, in a timely way, efficient, effective, and equitable allocations of health resources. The choice of cohort type (see Table 1) is dependent on the conditions and exposures being studied and ranges from the light-touch, long-term open cohorts capturing demographic indices (e.g., health and demographic surveillance system) to highly detailed cohorts created for specific purposes such as the genomics of a particular health condition. Some may need to be large enough and sufficiently powered to detect moderate effect sizes for rarer exposures and conditions (such as some disease- and exposure-based cohorts), and others may require very detailed data capture over a particular period of the life course (e.g., birth cohorts or cohorts of the elderly) to be able to explore the drivers of particular conditions affecting that subgroup or impacting future health.

There is a particular strength with multicountry cohorts and networks in addressing issues of generalizability or differing exposures and biology; however, they bring their own challenges. Retrospectively networked cohorts, often created opportunistically, face challenges with standardization, and on the other hand, prospectively networked cohorts are expensive to establish and face governance challenges with ownership and leadership.

Since this review has a specific focus on longitudinal population cohorts, their purpose and extraordinary value are elaborated on in the next section.

## 3 The Value Proposition For Longitudinal Population Cohorts

In LMICs, particularly within Africa, the robustness of health systems and their adaptability to new and existing health threats are of paramount importance (8, 9, 26–28). Longitudinal population cohorts are a fundamental tool in comprehending these health dynamics and offering significant benefits that enhance health research and inform public health policy (29–32).

**Figure 2:**
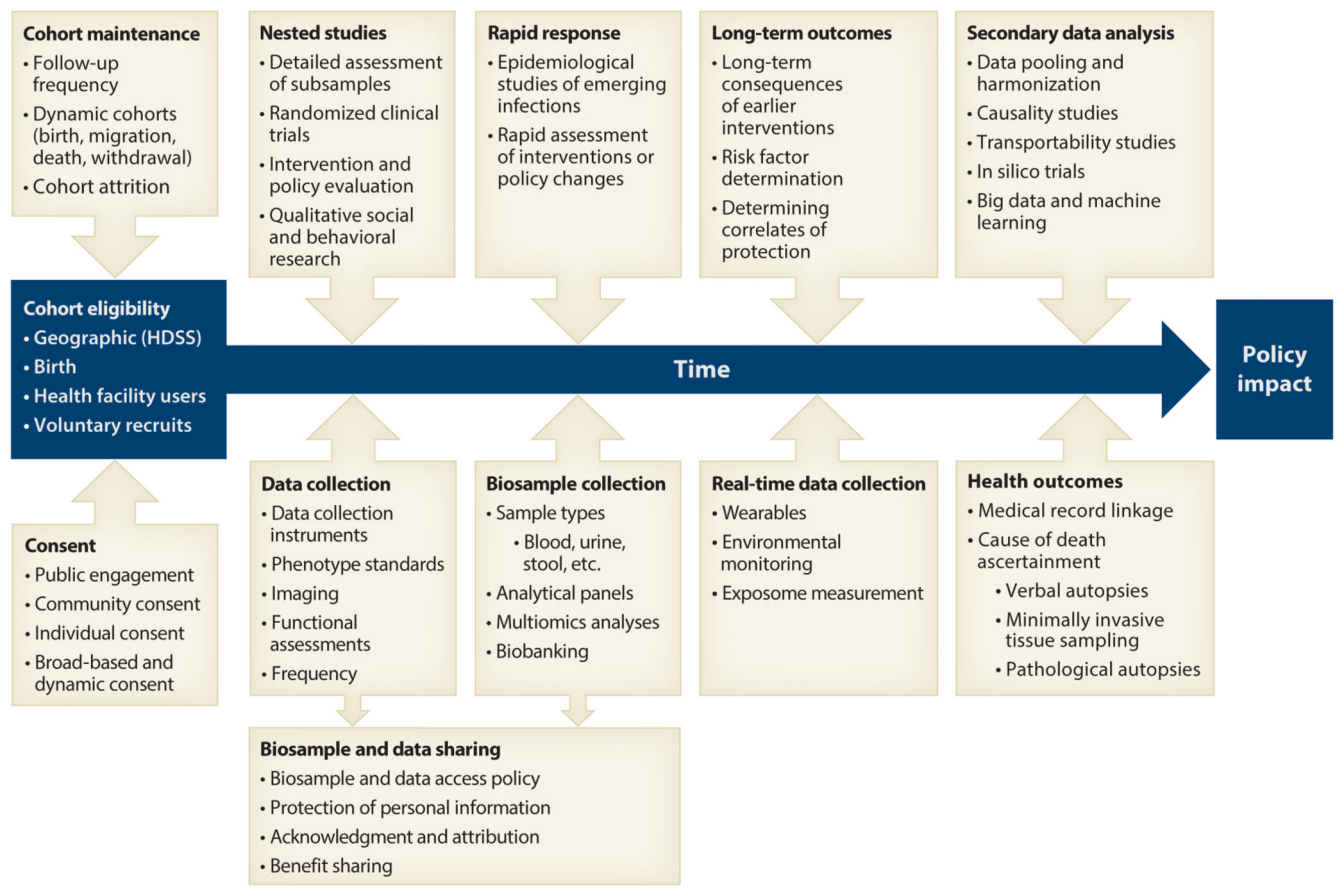
Value proposition of longitudinal population cohorts. Abbreviation: HDSS, Health and Demographic Surveillance System.

The core purpose of longitudinal population cohorts is to observe changes over time (Figure 2), documenting the sequence of events and timing of repeated measurements, exposures, and/or interventions. A classic example is a longitudinal study on childhood asthma (33–35). This temporal dimension is crucial for unraveling causality in multifactorial health determinants (36, 37). In Africa, where infectious diseases coexist with a growing burden of noncommunicable diseases (38), longitudinal cohorts can significantly contribute to understanding the complex interplay of environmental, behavioral, and genetic factors determining health outcomes (39).

Furthermore, longitudinal cohorts permit deep phenotyping of participants as seen in projects such as the UK Biobank (40), the China Kadoorie Biobank (41), and the All of Us initiative in the United States (42). In the African context, this advantage allows the incorporation of additional measures as newer technologies (43) become available or as funding is secured, thus enhancing the comprehensiveness and depth of data (44).

The enrollment of participants into a longitudinal cohort and ongoing community involvement foster improved participant engagement (45–47). This engagement translates into sustained trust, loyalty, and increased participation rates, thereby minimizing loss to follow-up. In Africa, such active participant involvement has the potential to facilitate a citizen science approach (48, 49), augmenting the impact of the research on participant well-being, as well as fostering a research culture (50).

Longitudinal population cohorts offer several advantages when serving as sites for clinical trials. Firstly, deep phenotyping of the cohort enables the precise identification of the study population and stratification according to different factors such as risk level or disease stage. This enhances the efficiency of participant recruitment and can lead to more representative and generalizable trial results. Secondly, longitudinal cohorts are characterized by their long-term follow-up of participants, capturing data at multiple time points. This continuous data collection can be valuable for trials as it allows the observation of the temporal progression of disease and the long-term effects of interventions (51). Thirdly, the relationships established with the community and the trust built among the cohort members facilitate participant engagement and retention in clinical trials, reducing dropout rates and enhancing data integrity (45).

Longitudinal cohorts also serve as excellent intervention research infrastructures. These cohorts provide a comprehensive understanding of the multifactorial determinants of health outcomes within the community, thereby informing the development of contextually appropriate interventions. Moreover, the existing infrastructure of a cohort, including data management systems, trained personnel, and established community relationships, provides an ideal platform to implement and evaluate interventions. Such infrastructure accelerates the intervention research process, reducing the cost and time associated with setting up new systems (52).

Longitudinal cohorts provide flexibility, permitting rapid response to emerging health threats. This feature was exemplified by the pivot to COVID-19 studies by several cohorts during the pandemic (53, 54). The COVID-19 pandemic also illustrated the usefulness of longitudinal cohorts to develop detailed infectious disease transmission models (55).

The value of longitudinal cohorts is also enhanced by record linkage to service records, such as electronic medical records (EMRs) (56, 57). Although establishing EMRs is more challenging in Africa due to low penetration, investment in these records for cohort members could catalyze wider EMR adoption (58).

Accumulated longitudinal data are also an indispensable resource for training in epidemiology, demography, and biostatistics (59, 60). The multifaceted data coupled with outcome measures, detailed phenotype data, health service utilization, and omic data allow for unprecedented discovery potential using traditional data analysis techniques as well as machine learning approaches.

The necessity of long-term investment in establishing a cohort encourages the standardization of study procedures and data structures, enhancing harmonization efforts across different cohorts, such as H3Africa phenotype standards (25) and Global Alliance for Genomics and Health initiatives (61). The harmonization and standardization inherent in longitudinal cohorts enable the transportability of research findings across different settings, thereby informing policy on a broader scale (62, 63).

Data sharing between cohorts comes with the inherent challenges of navigating legislation on protection of personal information, data ownership, and other issues across multiple national borders. It is a topic that deserves its own review and faces several challenges. First, the contributions of individual researchers and data managers in maintaining and supporting cohort data curation must be recognized. Second, with highly detailed data sets and longitudinal data, there are specific challenges with adequate anonymization to prevent the inadvertent or malicious identification of individuals and protect privacy. Historically, anonymization has been confined to the removal of direct identifiers and locators, such as names and residence (through address or geographical coordinates), but little attention has been given to the potential scope for an attacker to deduce identity through indirect identifiers such as age, sex, occupation, educational level, or attendance at a particular clinic, for example, combined with event date data. This is an emerging field of research (64) for data scientists in Africa, and acceptable disclosure risks with preserved utility have been achieved with the application of varying noise levels to status and time-varying variables on HDSS data. Adequate anonymization is closely linked to the requirements of protection of personal information legislation that are increasingly being adopted by African countries. Third, existing electronic data collection software is optimized for either cross-sectional studies or clinical trials and requires enhancement to be suitable for the highly complex and integrated data in longitudinal cohorts. Although standards exist for phenotypic and genomic data (65, 66), the development of adequate data models for cohorts is still in its infancy.

Longitudinal population cohorts distributed across the African continent have the potential to facilitate in-depth analysis over extensive temporal spans to elucidate the dynamic interplay between fluctuating environmental conditions and diverse health outcomes (67). Leveraging longitudinal data sets from these cohorts allows for evaluation of climate-sensitive health determinants and outcomes, including the prevalence and incidence of infectious diseases, noncommunicable diseases, and nutritional deficiencies attributable to evolving climatic patterns (68). Furthermore, these longitudinally maintained cohorts enable the research community to develop robust evidence bases that are important for the formulation and implementation of adaptive, mitigative, and preemptive strategies aimed at ameliorating the adverse health effects of climate change. Although by their very nature population cohorts are often geographically restricted, they can provide important triangulation and insight into nationally or regionally less detailed but representative data sources. As the importance of nontraditional data sources, such as remote sensing or mobile data, increases through the use of machine learning and other big data methods to address information gaps on the impact of climate change on human health and well-being, population cohorts organized into consortia such as the proposed African Population Cohorts Consortium (APCC) can provide important ground truths for the validation of such models, enhancing the trust of decision makers in these data sources and improving the likelihood of policy impact.

While these benefits are substantial, the expense associated with maintaining longitudinal cohorts is considerable, often making funders hesitant to commit to their long-term funding. To overcome this, government support is essential to recognize these cohorts as national research infrastructures that can attract study-specific external funding (52). Partnerships with private enterprise can also contribute to a sustainable funding model with carefully negotiated governance frameworks.

## 4 Uilding Cohorts Across Africa

Africa needs large population and disease-centered cohorts because the origin of much of the emerging disease burden in Africa goes against the paradigms developed in high-income countries, largely in the global north. Unpicking the contradictions and understanding when and how to intervene to prevent long-term morbidity are key to developing policies for population health (69). This process requires research and surveillance cohorts with large sample sizes and longterm follow-up. Many have argued the benefits of such cohorts, for example, the Comprehensive Health and Epidemiological Surveillance System (70) and the African Biobank and Longitudinal Epidemiological Ecosystem (71).

Health data from Africa are often cross-sectional in nature and highlight the burden of disease without providing possible explanations or hypotheses that involve complex interactions between different environmental factors. Understanding health trajectories and where and how to intervene to reduce adverse health impacts of the emerging burden of long-term conditions requires long-term studies and careful phenotyping (72).

The number of successful continental African cohorts with at least 10,000 participants, or 2,000 if they are birth cohorts, has increased over the past decade as more funding has become accessible to the African research community. The proposed APCC developed a scoping document that summarizes the present landscape of cohorts across Africa following a meeting in Uganda in March 2020, which involved seven funder organizations and many African investigators (73). In this section we highlight success stories and comment on the different funding models to support the sustainability of cohorts in an African setting.

An HDSS is a specific type of longitudinal population cohort that follows all the members (irrespective of age) of households resident in a defined geographic area, resulting in a dynamic cohort with inflows resulting from births and outflows resulting from deaths and out-migration (24). Historically, HDSSs appeared in the second half of the twentieth century, responding to a dearth of accurate population data in poorly resourced settings to contextualize the study of interventions to improve health and well-being (31). Since then, HDSSs have contributed to expanding our knowledge of a wide range of health and well-being interventions and outcomes.

Traditionally, many HDSSs have not collected biological samples, but this scenario is changing with the development of biobanks across Africa. A notable example is the H3Africa Consortium (https://h3africa.org/), initiated in 2012 and funded by the US National Institutes of Health and Wellcome Trust in partnership with the African Society of Human Genetics and the African Academy of Science. H3Africa supported the development of three regional biobanks, in Nigeria, Uganda, and South Africa. The aim of H3Africa was to build capacity for genomics and bioinformatics across African countries and to support African-led development of research ethics and data sharing guidelines to support projects and biorepositories (25, 74). Although many of the projects are disease centric, addressing common and high-burden diseases such as sickle cell anemia (75), chronic kidney disease (76), stroke (77), and glaucoma (78), there are two that fulfill the criteria for a population cohort: the Africa Wits-INDEPTH partnership for genomic research (AWI-Gen) (79) and the African Collaborative Center for Microbiome and Genomics Research (ACCME) (80).

AWI-Gen started as a population cross-sectional study of approximately 12,500 adults (mostly 40 to 60 years of age at baseline) to explore environmental and genetic contributions to CMDs in four African countries (Burkina Faso, Ghana, Kenya, and South Africa) (79, 81). AWI-Gen has been expanded into a longitudinal cohort with a second wave of data collection approximately 5 years from baseline. At baseline, AWI-Gen participants had high prevalence of several CMDs, including hypertension, chronic kidney disease, and diabetes, with considerable geographic and sex-specific differences. These findings require further study to better understand risks and trajectories of disease in different African settings. There are important genetic population substructures across the AWI-Gen study communities, and even within the three South African study sites (82), that need to be considered in genetic association studies and when exploring gene-environment interactions. AWI-Gen has published many genome-wide association studies (GWASs) for cardiometabolic traits and end points and has explored the potential transferability of polygenic scores developed in Eurocentric and multiancestry studies, which generally show poor performance.

ACCME is a Nigerian prospective longitudinal cohort study of approximately 11,700 adult women (80) that has also collected data on general and cardiovascular health.

As a partnership between several H3Africa research studies, the Cardiovascular H3Africa Innovation Resource was developed to harmonize and jointly analyze data related to cardiovascular health (83). The first analysis investigated the relationship between obesity and hypertension, showing regional differences and highlighting that obesity doubles the risk for hypertension, and therefore presents an opportunity for targeted intervention (84).

The transcontinental H3Africa Consortium, involving 30 African countries, supporting over 50 projects, and having published over 700 manuscripts over a 10-year period, is now planning its next steps. As the major dedicated funding has come to an end, many of the cohorts are facing an uncertain future (85). H3Africa has, however, made provisions for the secure storage of data in the European Genome-Phenome Archive and for responsible sharing of data and samples through the H3Africa Data and Biospecimen Access Committee, which is now hosted by the Science for Africa Foundation (https://scienceforafrica.foundation/). Fortunately, some cohorts have been successful in securing additional funding, but many will no longer collect longitudinal data.

There are also other large African cohorts with genomic data. The Uganda Genome Resource (UGR) (19) is a substudy of the Uganda General Population Cohort which was established in 1989 (86), and UGR currently has data on genome-wide genotyping of approximately 5,000 individuals and whole-genome sequence data on approximately 2,000 individuals. Research on the UGR has demonstrated considerable population substructure in Uganda, and GWASs have been performed for a wide variety of common diseases and traits, as well as polygenic score analyses and comparative studies with other populations worldwide, with the aim of assessing cross-ethnic transferability, which has been shown to be limited (87). The Non-Communicable Disease Genetic Heritage Study consortium aims to provide insight into genetic diversity across Nigeria and to examine the burden of disease and genetic associations with noncommunicable diseases in Nigeria (18).

**Figure 3:**
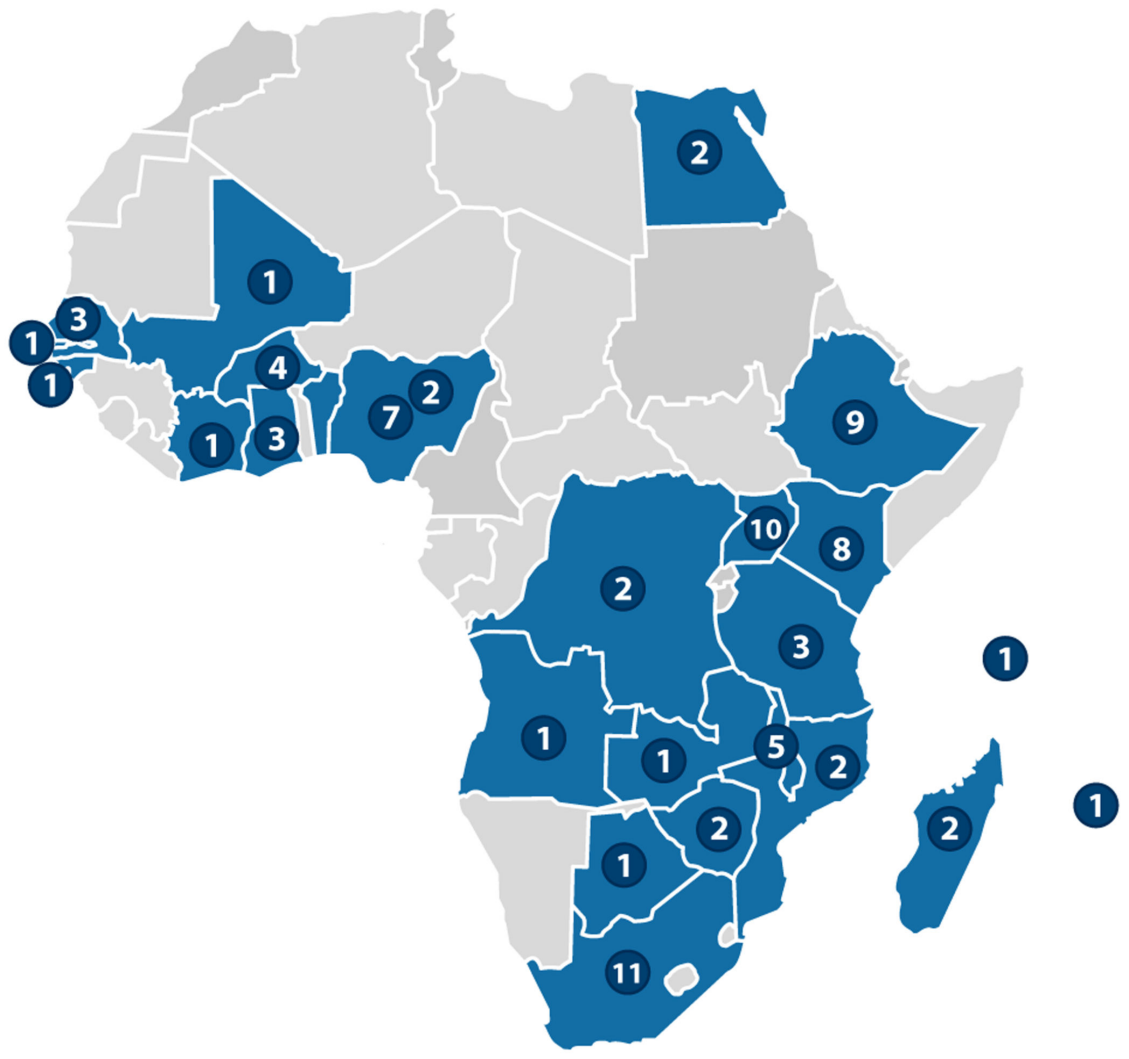
Location of current cohorts in Africa. The numbers denote the number of cohorts in specific countries.

As part of a scoping exercise by the Collaboration for the Establishment of an African Population Cohorts Consortium, ongoing or recently completed cohorts in Africa have been identified and characterized (summarized in Table 2, with details at https://ce-apcc.org/cohorts). Only longitudinal population cohorts surveying at least 10,000 individuals (or 2,000 if they are birth cohorts) are included, irrespective of whether they currently collect or store biospecimens. The most common type of cohort is part of HDSS sites (~63%), and Figure 3 shows the countries in which cohorts are based.

## 5 Obstacles To Establishing And Sustaining Cohort-Based Research In Africa

There are particular obstacles in Africa compared to other settings (Table 3), both in the establishment of country-specific cohorts and in the comparison and harmonization of data between countries with highly diverse populations and widely varying governance procedures. These obstacles prevent meaningful comparisons and conclusions at a continental level. Establishing and sustaining population cohort–based research is challenging in any setting, and there should be clear objectives and stakeholder engagement from the outset to justify not only the resource commitment but also the participant burden.

Despite these challenges, we have shown that there are multiple high-quality and productive long-term cohorts and networks, which is a testament to the commitment and innovativeness of the institutions and individuals involved. However, long-term sustainability of cohorts in Africa is often hard to achieve, due in many cases to (*a*) resource constraints, with the bulk of funding in low-income settings being externally derived and related to specific research questions with timelimited funding; and (*b*) marked political and economic instability. There are thus large groups of highly vulnerable and mobile individuals who are frequently undocumented, as they move between or within countries, seeking refuge or economic opportunity, and are largely excluded from longterm studies.

The paucity of national resources also impacts the availability of reliable civil registration (88) and health service information systems (89), on which wealthier countries rely, to provide reliable and consistent denominators and sampling frames and exposure and outcome data (90). Additionally, participants may be less likely to be available by postal or telephone services, exacerbated by high levels of functional illiteracy. This leads to a reliance on more costly face-to-face selection, data collection, and follow-up and capture.

There is a role for local investment through national governments identifying priorities and being supported by regional organizations such as the African Union, Africa Centres for Disease Control and Prevention, and World Health Organization Regional Office for Africa.

## 6 The Future Of African Cohorts For Health And Well-Being

Research culture in Africa is diverse and evolving, and this influences both existing and future cohorts and their impact on health-related research. It varies across countries and regions due to factors such as historical influences, resources, and government support, and key aspects of research culture in Africa are outlined in Table 4. Overall, the research culture in Africa is dynamic, with both challenges and opportunities for cohort-related research, and efforts to strengthen research infrastructure, increase funding, and promote collaboration are essential for its continued growth and impact (91).

Having a coordinated vision for developing existing cohorts and creating new cohorts across Africa will greatly enhance the understanding of disease prevalence, access to health care, and biological pathways to health, as well as opportunities for comparative analyses. Achieving such a vision will require joint decision making to enhance interoperability across cohorts and to develop data resources that can more easily be accessed and shared, both within the continent and across the world.

As mentioned above, in an effort to link multiple cohorts across Africa and to develop a blueprint for existing and future cohorts in Africa, the formative phase of the APCC is currently underway, led by groups from Kenya and South Africa and with continental representation among its leadership. This will be important in developing communities of practice to enhance cohort research across Africa.

There is a unique opportunity to study the interaction between infectious diseases, noncommunicable diseases, and the broad environment, including climate change in African communities. Developing longitudinal population cohorts provides an opportunity for data harmonization and the development of continental data and sample resources with sound governance principles that will permit ethically sound and legally compliant data sharing and use. This can help African governments increase their awareness of common noncommunicable and infectious diseases and shift the focus from disease management to prevention and cure.

In closing, developing and enhancing coordinated research and surveillance cohorts across Africa has great potential and will lead to new knowledge on the complex interaction of susceptibility to disease and multifaceted environments. As the research and funding communities pivot to global health perspectives, national ministries of health will have more data and evidence to develop interventions and identify focus areas to promote the health and well-being of their communities and populations. A single one-size-fits-all approach does not work in the context of improving individual and population health, and opportunities for cross-learning among African countries will be enhanced though the development of coordinated cohorts. Population cohorts and patient-centered hospital cohorts will make a difference in Africa and for the African diaspora and, while addressing health disparities, will lead to discoveries that will be relevant to global populations.

## Figures and Tables

**Table 1:** Types of cohorts and networks, highlighting strengths and limitations in the African context

Cohort type	Description of typical cohort	Purpose(s)	Strength(s)	Limitation(s)
**Single-site or single-country cohorts**				
Long-term birth cohorts	Recruitment from pregnancy, birth follow-up, through childhood and beyond May be nested in HDSS or population cohort, often with linked biobanks	Life-course epidemiology Usually related to particular outcomes/exposures Developmental research	High level of detail Capture data from early life and have frequent follow-up Potential to recontact later in life	Dropout leads to bias May not be representative High cost
HDSSs	Geographically defined, total population capture, open cohort Vital events captured Migration patterns captured	Demographic indices Population sampling frames	Large cohorts Usually followed over decades Opportunity for nested studies	May capture demographic episodes rather than track individuals Long-term projects may lead to less representativeness Urban HDSS is challenging
Service-based cohorts	Collects data on service users from a health facility with defined catchment area	Clinical data and disease progression	Low cost Focused	Dependent on service usage Catchment area may be undetermined
Exposure cohorts	Tracking of individuals with specific exposures, for example, occupational cohorts or cohorts of urban versus rural dwellers and air pollution	Understanding the risk of a wide range of outcomes associated with different exposures	Opportunity to explore the biology of exposure	May not capture useful data on health, behavior, and other exposures
Disease cohorts	Tracking of individuals with diagnosed disease (either through presentation to services or through active screening and recruitment)	In-depth understanding of disease biology and progression/prognosis	High level of detail on specific diseases Often good retention as linked to clinical care Can capitalize on routine data sources	Limited to specific health conditions May not capture relevant related data
Population cohorts	Repeat observations on defined population, often linked to HDSS, often with linked biobanks	Capturing prevalence and incidence of common conditions and behaviors	Broad range of conditions Good understanding of life-course trajectories	High cost
**Networks or multicenter cohorts**				
Post hoc data pooling	Pools data from existing cohorts or HDSS for comparative or larger analyses	Sharing of data from existing cohorts for specific research questions	Low cost Focused	Challenges with protocol and variable harmonization Data-sharing complexities
Prospective data collection	Multisite/country cohort initiatives usually with a specific aim or disease focus	Establishing a multicenter cohort for a specific purpose	Standardized protocols and tools across all sites	High cost Autonomy of individual institutions challenged Governance and ethical approval complexities

Abbreviation: HDSS, Health and Demographic Surveillance System.

**Table 2:** An overview of the number and nature of ongoing or recently completed cohorts in Africa

Region	Type of Cohort			Total
	Birth cohort	Cohort	HDSS	
North Africa		2		2
West Africa		5	16	21
Central Africa		1	2	3
East Africa	1	10	25	36
Southern Africa	2	7	9	18
Multiregion		3		3
Total	3	28	52	83
Individuals under observation	17,300	429,670	5,009,260	5,456,230

Abbreviation: HDSS, Health and Demographic Surveillance System.

**Table 3:** Key differences between high-income and low-income settings that impact the success of longitudinal population cohorts

Requirement	HIC experience	LIC/Africa experience (several may apply to each setting)
Political stability	Stable government and infrastructure	Frequent political instability and fragmented infrastructure
Stable populations	Stable, documented populations	Vulnerable highly mobile groups
International frameworks	Frequently established regional frameworks and governance systems for collaboration and data sharing	Ad hoc study-specific arrangements
Secured funding	Government contributions and well-resourced funding organizations	Donors/research funding organizations Additional expenses: participant remuneration, heavy human resource needs, transport for tracking participants (expensive to establish and maintain)
Recruitment of participants	National databases/census	Geographically defined: issues with representativeness, urban and cultural challenges
Follow-up with participants	Internet or postal surveys, screening programs	Face-to-face interviews, transport, out-migration from population
Denominator data sources	CVRS	CVRS lacking or incomplete
Capturing health and vital outcomes	Electronic medical records, health management information systems, disease registries, postmortems, and death certification	Manual, patient-held records; patchy EMRs + HMISs + DRs (and diseases undiagnosed or misclassified) Verbal autopsies to assess cause of death Limited diagnostic capability
Capturing exposures (via databases, surveys)	Rich electronic data sources (insurance, GP and hospital settings), participant-completed postal and electronic surveys	Scant electronic data sources Interviewer-led face-to-face questionnaires Literacy issues
Research infrastructure and expertise: administration, data management, biorepository, internet, utilities	Wealth of infrastructure and available expertise	Fragmented infrastructure Specialists and experts overstretched (particularly in remote/rural locations)
Trust in research institutions	Mixed level of trust, but generally good High potential for infodemic	Mixed level of trust, but generally low Belief systems may be incompatible with scientific approaches to disease causality High potential for infodemic

Abbreviations: CVRS, civil and vital registration systems and statistics; DR, disease registry; EMR, electronic medical record; GP, general practitioner; HIC, high-income country; HMIS, Health Management Information System; LIC, low-income country.

**Table 4:** Key aspects of research culture in Africa

Factor	Explanation of impact
Growth and investment	Many African countries have been increasing their investments in research and development, fostering a growing research culture. Research institutions and universities are expanding their facilities and collaborations.
Challenges	Despite progress, Africa faces challenges such as limited funding, inadequate infrastructure, and brain drain. These factors can hinder research activities and the retention of talented researchers.
Multidisciplinarity	African research often focuses on addressing pressing societal issues such as health care, agriculture, and sustainable development. As a result, multidisciplinary research is common.
Collaborations	Collaboration between African researchers and international institutions is vital. Partnerships with universities and organizations from other continents contribute to knowledge exchange and capacity building.
Indigenous knowledge	African research culture often seeks to integrate indigenous knowledge systems, recognizing their importance in solving local challenges.
Capacity building	Efforts to train the next generation of African researchers are ongoing. Scholarships, mentorship programs, and academic exchange programs play crucial roles in capacity building.
Publishing and journals	African researchers contribute to academic journals, with some countries having their own reputable publications. Open access initiatives are gaining traction to increase the visibility of African research.
Policy influence	Researchers in Africa increasingly influence policy decisions at the national and regional levels, contributing to evidence-based governance.
Language diversity	Research is conducted in various languages, with a focus on both local languages and English, French, and Portuguese for wider dissemination.
Regional variation	Research culture can vary significantly between North Africa, West Africa, East Africa, southern Africa, and central Africa due to distinct historical, linguistic, and socioeconomic factors.
